# Some Observations on the Cutaneous Eruptions Attending the Use of Chloral

**Published:** 1880-09

**Authors:** 

**Affiliations:** Interne des hôpiteaux


### Original Translations.

### ARTICLE VII.

### SOME OBSERVATIONS ON THE CUTANEOUS ERUPTIONS ATTENDING THE USE OF CHLORAL. By Mr. MAYOR, Interne des hôpiteaux. A Communication to the Clinical Society of Paris.

[Translated for THE CHICAGO MEDICAL JOURNAL AND EXAMINER by H. D. VALIN, M.D., Chicago.]

In 1874 we had, for the first time, an opportunity to observe a patient under the care of Prof. Vulpian, who presented every day, at about the same hour, a peculiar eruption on various parts of his body. Our Professor did not hesitate to refer these symptoms to the use of the remedy which formed the basis of the treatment, that is, chloral. Paulin (Eugene) came under the care of Dr. Vulpian at La Pitié in 1873, suffering very probably from transverse myelitis, but presenting somewhat peculiar phenomena. They were true tetaniform fits, with opisthotonus, emprosthotonus and trismus. These attacks, to which he is yet subject at times, although in a better condition now, were extremely frequent in 1874. We then used to give him repeated doses of chloral until he was relieved. In this manner he would take as much as fourteen grains of chloral in the twenty-four hours, and, during the following days, we had often to renew the administration of that medicine in smaller doses, whenever the patient presented some convulsive shakes or some contractions. It is in these conditions that the eruptions of which we will speak appeared.

We find on record, on June 16, the following facts: From the beginning of May, patient noticed on his hands and face the appearance of small red spots, fading away after half an hour. From

May 10th he had, every evening at about five, pricking of the face, the top of the hand, the wrists, the knees and the region of the sternum. Raspberry red spots would next appear on these parts, with fine elevations, the size of a millet-seed. These spots used to disappear at 8 p. m., but the next day one could see traces of them in the shape of a light erythema, accompanied with itching. During the eruption patient would feel oppressed, and the pulse was very rapid. On the evening of June 15, after patient had taken his fourth grain of chloral, he was taken with violent fits of coughing. His sputa was streaked with blood, and he vomited. The 16th, in the morning, same sputa. June 17th, two very marked eruptions, with cephalalgia, oppression and nausea, followed by sleep. At 5 p. m. new eruption, even considerable, consisting in some large spots on the knees, the legs and the feet, a patch on the right arm, and a few spots on the right side of the face. Simultaneously, considerable oppression and acceleration of the pulse (100).

Then we stopped the administration of chloral, notwithstanding which the eruptions, accompanied with dyspnœa, continued till June 20th. On the evening of that day there was some dyspnœa till 8, soon followed by the apparition of a single spot on the right wrist. June 21st, same hour, eruption more marked. During the last three weeks, daily epistaxis in small quantity.

About the middle of July he had to resume the chloral on account of a tetaniform attack; accordingly, the same cutaneous and respiratory symptoms appeared as early as the second day. An important thing to note is, that the man who had unilateral sweats confined to the left side of his body presented eruptive phenomena on both sides alike. The fact of a cutaneous eruption resulting from the use of chloral is nothing particularly new. According to Mr. Gubler* Wilkie Burmann has noticed the scarlatiniform rash, accompanied with inflammation of the mucous membranes. Schüle and Kirn also describe it. Other authors have observed purpura and uricaria, but in connection with signs of a high degree of poisoning. The orthopnœa has been noticed by several observes, among which Mr. Bordier.† More than one clinical observer could make similar observations.

*Gubler. Nouveaux commentaires du codes.

†*Journal de Therapeutique*, 1877.

But what strikes us is the course, almost always regular, of the symptoms in the cases which fell under our observation since. Here is a record of them:

CASE II. P. (Marie), aged 52, came under the care of Dr. Bernutz on account of a peculiar floating abdominal tumor, which a puncture showed to be probably an ovarian cyst. This puncture was made on March 17, and the patient, complaining of sleeplessness, she was administered two grains of chloral. On May 7, P. said that for the last three weeks she had had a rush of blood to her head, and palpitation with oppression every day after supper. These symptoms returned regularly till July 18, although they could not be accounted for by any morbid state of the heart or lungs, nor by the size of the cyst, which was punctured once more without changing in any degree the fits of oppression. But, as these became more intense and lasting, I observed one of them on July 18 at 5 p. m. The patient, who lay in the dorsal decubitus, was extremely oppressed, and her pulse was strong and frequent (100). The sounds of the heart were not disturbed. Patient was in such throes that she almost lost consciousness. Her eyes were half closed, and it was impossible to elicit an answer from her, even through signs; she did not seem to hear. Her respiration was labored, in the manner of some asthmatic patients during a fit of dyspnoea. Auscultating the chest revealed nothing abnormal, but a bright rosy mask covered all the face. On the arms, the forearms, the superior parts of the chest, the anterior parts of the knees, and the instep, bright rosy spots, irregular circular, slightly corrugated at the surface, not very elevated above the sound skin, but presenting sharp but somewhat sinuous edges appeared. The administration of chloral was immediately stopped.

The next day, July 19, the parts formerly occupied by the rash were the seat of a furfuraceous desquamation more marked in some places than in others.

July 23. This evening patient tried to eat a little, which she had not done, except in the morning, since her fit on the 18th.

As before, the meal brought the recurrence of the symptoms just described, which had not appeared for the last few days.

On August 2 patient left, without having been able to take any meal in the evening since July 23. Neither the eruption nor the accompanying troublesome symptoms returned.

Two points in this case seem to us important: first, the evident relation of the appearance of the rash with the time of gastric digestion, and the absence of auscultative disturbances during the fit of dyspnœa.

CASE III.—X., aged 27, suffering from tuberculosis of the bronchial ganglia, with compression of the trachea and bronchi, came under the care of Dr. Le Dentu at St. Antoine.

On Feb. 10 the patient, who had been taking daily two grams of chloral at a dose for ten days, complained of flushing of the face, with increase of her habitual dyspnœa; and moreover, she noticed yesterday for the first time red patches on the extensor side of her forearms. These patches were the seat of some sharp itching; yet, this morning, there was no desquamation on these points. Chloral was discontinued.

Feb. 11, almost no remains of the sensation of warmth. Patient left for the Vesinet.

CASE IV.—L. (Fanny), aged 44, had been operated on for the removal of an epithelioma of the left breast, Feb. 13, by Dr. Le Dentu. During the following fortnight she did not take any chloral. She said, nevertheless, that she had some flushing of the face after meals.

On the evening of March 11 she took one gram of chloral hydrate. As early as the next day, after breakfast, a few red points broke out on the back of her right hand. On that evening (March 12), she took two grams of chloral.

March 13. Flushing of the face, without any dyspnœa yet. Following the redness of the face, redness on the whole of the inferior half of the right forearm and the back of the hand. Two grams of chloral in the evening.

March 14. After dinner, which is her chief meal, patient had flushing, with redness of the face and redness of the forearms, more marked on the right, and no itching. But, as she felt a little itching on the feet, she noticed small red points on their dorsal aspect.

She was taken at the same time with a peculiar uneasiness and some dyspnœa, with the sensation of a bolus in her throat. She remained quiet, without uttering any word, nor did she lose consciousness, but she wept during half an hour. This woman had never shown any sign of hysteria. She took no chloral that night, and the next day there was redness of the face only; but as she resumed the remedy on the following day, in one to two gram doses every evening, the annoying symptoms returned, and on March 19 at noon, that is half an hour after meal, I could notice the following fact: her face was uniformly red, except the chin, above which the redness ended in a somewhat elevated border. There was no sharp edge elsewhere. On the back of the hand, skin glossy, but bright rose-colored. This hue, however, was not quite uniform. Three fingers'-breadth above the wrist the redness stopped in a sinuous border, not very distinct from the sound skin, for the hue faded as it receded from the wrist. The redness did not extend so high up on the left side, where it broke less rapidly. On the palm the redness was less intense, and did not ascend above the wrists. Slight rosy hue on the superior part of the chest, and very bright redness, without alteration of the surface of the skin, on both knees on the extensor side. The erythematous patches were wider than the hand in these places, and had no well-defined borders.

From the time the eruption appeared, there has been flushing of the face, with palpitation. The accelerated beats of the heart were so violent as to raise the shirt and the sheet. When I could examine the patient they were less violent, though very frequent (140). There was no alteration in the tone of the heart sounds, and no murmur. The dysponæa was hardly noticable. Chloral was discontinued.

March 20th.—Redness of face, slight palpitation. A few red spots on the top of the hand.

March 21st.—Some redness of face. No other symptom.

We will observe, respecting the patient, this fact alone: That the eruption constantly predominated on the right side; at least in regard to her arm. According to the patient, however, both her hands were a little clammy, and equally flabby. Should we then attribute this predominance to some slight injury to the nerves, caused by the fact of our reaching very deep into the axilla to remove a degenerated ganglion? Then we will observe that we do not understand why the eruption did not appear on the internal surface of the arm, where sensitiveness was much diminished in the region of the accessions. There was not any disturbance of sensation any where else.

CASE V.—L. Fernelly was suffering from an abscess of the vulvo-vaginal gland and a sanguineous cyst of the true pelvis, which opened into the bladder. This patient came under the care of Dr. Le Dentu, Feb. 18. On March 30, she was ordered a dose containing two grains of chloral. Less than eight days after, although she had not taken the whole dose every day, as she declared, she was taken with flushing of the face and some palpitation after meals. These symptoms went on increasing gradually, and as early as April 10th the patient had palpitation, oppression, redness of the face, and an eruption on the back of the wrists, and the anterior surface of the knees. But there was at the same time some gastric disturbances. Accordingly, chloral was ordered in a rectal injection on April 12th, and three grains were thus given. On the 13th, 4 grains were given. The 14th 5 grains. On examining the eruption on that day, it had the following character: Patient was seized immediately after meals with a sense of weakness and dizziness; the blood rushed to her face, and at the same time she felt violent palpitation, with dyspepsia. Her face was almost entirely red, and the skin glossy. So were her neck and upper part of chest. The hue was equally bright rose colored without elevation of the skin on the palmar aspect of the hands and fingers, and the soles of the feet. But we could notice at the same time red spots with sinuous borders, and an irritated skin on the dorsal aspect of the wrists, of the elbows over the olecranon, on the tops of the feet and the instep—on the kness over the patella. There was not the slightest itching.

Her pupils seemed rather contracted during the crisis. At any rate they were not dilated. Auscultation revealed no abnormality of lungs. On account of the excessive pain which the patient felt on a line with the hypogastrium we had to continue the injections of chloral in 5 grain doses every day.

But on April 22d a new incident occurred. She showed signs of a much more profound degree of poisoning: Skin cold, hyperæsthetic region here and there (on the body, particularly the back), marked pallor of face, extreme weakness and faintness, a tendency to syncope, dread of imminent death, and considerable dyspnœa without its being accounted for by any thoracic phenomena. Chloral was stopped accordingly for a few days, after which it was resumed on various occasions without it giving rise to any serious symptom.

We can see in this case that whatever the way in which chloral is administered it gives rise to the same symptoms; it is obvious that they do not depend on cardiac troubles resulting from the direct action of chloral on the stomach and the resulting dyspepsia.

Finally, I might cite the case of a patient under the care of Dr. Lancereaux, who was prevented from sleeping one night by a feeling of warmth, after she had been taking from two to five grains of chloral daily for eight days. This record, for which I am indebted to the good will of Mr. Dupont, out-door attendant, says that on the next morning the patient presented an almost universal eruption, consisting of rosy lichenous patches, which were the seat of severe itching. One might doubt, however, the nature of that eruption, as it disappeared completely in three days, after assuming a bronze hue, and did not return, although the chloral treatment was continued.

In all of these cases we see the chief features of chloral poisoning. The truth is that whenever a rather excessive dose of chloral is taken through accident, the patients are comatose, presenting at the same time a marked acceleration of the pulse, stertorous breathing and congestion of the integuments, particularly of the face and neck. It is only in a later stage that the pulse becomes less frequent, the face pale, and that the respiration finally grows slower, and even stops.

In our recorded cases, the symptoms appear in a determined order, so to speak, and present themselves with the features of a certain degree of regularity, at first the redness of the face following each meal, accompanied with a slight sensation of drowsiness and even sickness.

Soon the acceleration of the pulse is so marked as to become annoying. If the treatment is persevered in, to these symptoms, gradually increasing, is added dyspnœa, which was slightly noticeable the preceding days, but which becomes so marked as to absorb the entire attention of patients, and be the cause of their complaints. Should we observe them at that point, we would notice that not the face alone is red, resembling wine dregs in color, but this tint extends over the neck, the palms of the hands and sometimes the soles of the feet. These red spots, more or less marked, are variable in extent, and appear with sinuous borders generally. In some places the patch slightly encroaches on the neighboring skin, presenting at the same time a granulated aspect; in other places it is glossy, but does not project. This eruption evidently has favorite seats: the back of the hand and wrist, the upper and front part of the chest, the articulations of the knee and of the elbow on the extensor side, the top of the foot and the instep. The eruption generally lasts but a short while—from half an hour to a few hours; it is often itchy. Last of all, we sometimes observe, on the following day, some slight scaly desquamation on the part which it has occupied.

This eruption is as remarkable on account of its time of appearance as for its objective symptoms. We see that it always returns immediately after a meal. The time at which the remedy is taken seems to us to have nothing to do with the time of the recurrence of the troublesome symptoms. These, we would repeat, are dyspnœa chiefly, and palpitation of the heart, which constantly precede or follow a return of the eruption. The accelerations of the pulse may attain 140. The pulse is strong and energetic.

This description establishes in nowise a resemblance between these eruptions, those that appear in the course of some grave diseases, and the exanthema of variola, rheumatism, diphtheria, etc. From their pathology we would even be more tempted to compare them with erythemas due to the administration of such medicines as arsenic, mercury and iodine. But the course of the eruptions caused by these, and their appearance itself, differentiate them well enough from those just described. The belladonna eruption even resembles them more by its appearance, as well as by some concomitant symptoms, viz.: the acceleration of the pulse. But as to the erythema caused by the oleo-resins, it strikes us with its analogy of seat and appearance to those depending on chloral. Here is what Dr. Bazin says of the eruption caused by the oleo-resins :

" If the eruption breaks out slowly and in different places in succession, it is limited at first to various points which seem to constitute its various seats, such are the wrists, the malleoli, the knees, the hands and feet. It is also in these regions that it is the more marked through its course, and the more reluctant to fade. In other cases the exanthem is developed in a brisk and lively manner, invading all the integuments at once, when it is generally accompanied with febrile symptoms of a greater or lesser intensity.

" The roseola caused by the oleo-resins generally breaks out as red or rosy spots, unequally elevated, round or irregular at the edge, without any projecting part, and fading away under the pressure of the finger ; some remain small and isolated ; others are so confluent as to give to the skin an almost uniform redness. These spots, more or less confined to the regions alluded to at first, have but an ephemeral existence of themselves, and fade almost as soon as the use of the drugs is stopped. In case these are continued, it generally extends and pervades the whole surface of the body. The itching, very moderate at first, becomes very painful and at times intolerable. The eruption may undergo at the same time important modifications in its aspects and character, the elements of which they consist losing their appearance of simple maculæ to rise above the skin, and become true papules, appreciable by the finger and eye. This phenomena is more especially observed in the favorite places, in which large red patches are found, terminating at the edge in a sort of wavy and projecting bunch."

What is the mechanism of the eruption excited by chloral ? Shall we admit here what Mr. Bazin affirms of copaiba, a direct action of chloral as it is eliminated by the cutaneous glands? Indeed, we know that it is generally admitted that this medicine, which is certainly eliminated in part by the lungs and by the renal secretion, is likewise eliminated by the sudoriparous glands. But we may answer to this that the patient who was the subject of our first observations had profuse perspirations, confined to the left side, and that he had nevertheless a bilateral eruption.

Should we refer it preferably to vascular troubles? Says Prof. Vulpian, in his lecture: "The action of chloral on the vasomotors is plain. The vessels of the web of a frog's foot are dilated, and all the mucous membranes in the mammiferæ are congested during the sleep induced by chloral. Although the eruption is accompanied by marked irregularities in the beats of the heart, yet we must say that clinical studies do not furnish very satisfactory explanations on that point.

---

CAUSES OF SICKNESS IN MICHIGAN.—Reports to the State Board of Health, Lansing, for the week ending July 17, 1880, by sixty-five observers of diseases in different parts of the State, show causes of sickness as follows:

| DISEASES OBSERVED. | BY OBSERVERS. | | DISEASES OBSERVED. | BY OBSERVERS. | |
|---|---|---|---|---|---|
| | *Number.* | *Per cent.* | | *Number.* | *Per cent.* |
| Asthma | 1 | 2 | Fever, intermittent | 58 | 89 |
| Brain, inflammation of | 3 | 5 | Fever, remittent | 38 | 58 |
| Bowels, inflammation of | 13 | 20 | Fever, typhoid (enteric) | 7 | 11 |
| Bronchitis | 27 | 42 | Fever, typho-malarial | 13 | 20 |
| Cerebro-spinal meningitis | 1 | 2 | Influenza | 8 | 12 |
| Cholera infantum | 31 | 48 | Liver, inflammation of | 1 | 2 |
| Cholera morbus | 46 | 71 | Measles | 14 | 22 |
| Colic | 1 | 2 | Neuralgia | 35 | 54 |
| Consumption, pulmonary | 42 | 65 | Pneumonia | 11 | 17 |
| Croup, membraneous | 0 | 0 | Puerperal fever | 4 | 6 |
| Diphtheria | 17 | 26 | Rheumatism | 36 | 60 |
| Diarrhœa | 57 | 88 | Scarlatina | 6 | 9 |
| Dysentery | 25 | 38 | Small-pox | 0 | 0 |
| Erysipelas | 15 | 23 | Tonsilitis | 17 | 26 |
| Eyes, inflammation of | 1 | 2 | Whooping-cough | 27 | 42 |

By the last column it will be seen that the most widely distributed disease was intermittent fever (ague); next to that, diarrhœa; next to that, cholera morbus; no cases of small-pox; scarlet fever in only nine per cent. of the localities.

HENRY B. BAKER,
*Secretarg State Board of Health.*

